# Deep Parotid Lobe Abscess Presenting with Dysphagia and Trismus

**DOI:** 10.1155/2019/2931015

**Published:** 2019-02-24

**Authors:** Madison Grinnell, Andrew Logeman, Timothy Knudsen, Zafar Sayed

**Affiliations:** ^1^University of Nebraska College of Medicine, Omaha, NE, USA; ^2^University of Nebraska, Department of Otolaryngology–Head and Neck Surgery, Omaha, NE, USA

## Abstract

An abscess of the deep parotid lobe is an uncommon complication of acute parotitis. Characterized by warm erythematous facial skin and ipsilateral cheek swelling, parotid abscesses have often been associated with decreased saliva production and immunodeficiency. We offer a case of a large deep parotid lobe abscess presenting similarly to a peritonsillar mass, causing significant odynophagia and difficulty swallowing. Computed tomography scan revealed an infected deep parotid lobe sialocele which was surgically drained transorally and treated expectantly with antibiotics.

## 1. Introduction

A variety of bacteria and viruses may cause acute infection of the parotid gland. The typical presentation of acute parotitis consists of sudden-onset erythematous, warm skin, and tissue swelling over one cheek. Patients often have a fever, leukocytosis, and tenderness in the affected area. Abscess formation is a possible but uncommon complication and is most often seen in elderly patients in the setting of dehydration, poor oral hygiene, or recent oral surgery. It is theorized that this is due to the potential for retrograde spread of oral bacteria into the gland via Stensen's duct under these circumstances [1]. Additional risk factors include a history of diabetes mellitus or Sjogren's syndrome. Abscess formation may occur through hematogenous spread or suppurative lymphadenitis of the intraparotid or periparotid lymph nodes. Here, we present a case of a deep parotid abscess with an unusual presentation.

## 2. Case

A 74-year-old man presented with a chief complaint of a right-sided sore throat with odynophagia. He was febrile and had decreased his oral intake due to difficulty and pain swallowing. He was admitted from the emergency room to the ENT service due to concerns of airway edema, right oropharyngeal swelling, and right parotid tenderness. Edema and swelling involving the mucosa of the right palatine tonsil, oropharynx, uvula, base of tongue, and epiglottis with additional edema in the right masticator space were noted on flexible laryngoscopy. The significant oropharyngeal swelling caused leftward deviation of the uvula as well as trismus that was initially worrisome for a peritonsillar infection.

**Figure 1:**
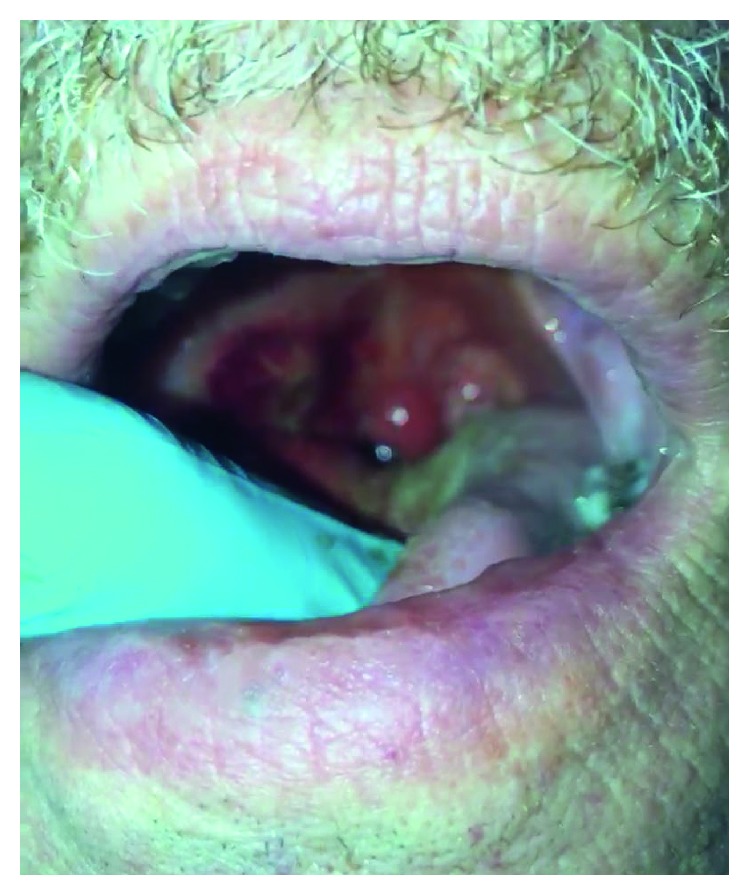
Exam demonstrating right oropharyngeal prominence.

**Figure 2:**
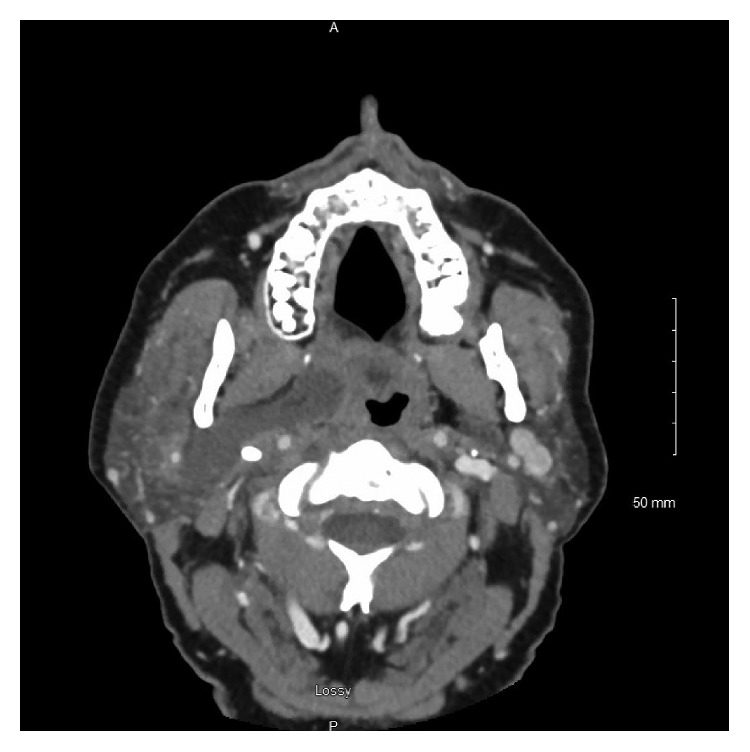
Axial CT image revealing retromandibular right deep lobe parotid abscess.

**Figure 3:**
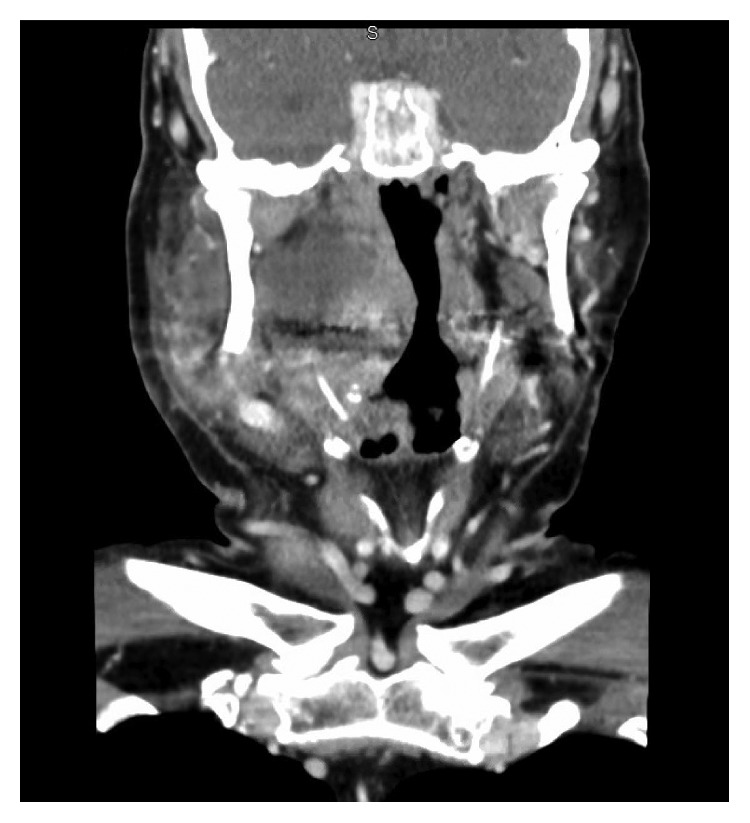
Coronal CT image depicting deep lobe parotid fluid collection extending to the prestyloid parapharyngeal space.

Ampicillin and steroids were given; however, he continued to have oropharyngeal prominence. Subsequently, an interval CT scan of the neck was obtained, revealing a hypodense deep parotid lobe sialocele measuring approximately 5.0 × 0.9 cm extending to the parapharyngeal space and exerting mass effect on the oropharyngeal airway. No prominent sialolith was noted. Several deep jugular chain lymph nodes in level II were also mildly prominent. The fluid collection was drained transorally via an incision lateral to the palatine tonsil along the anterior tonsillar pillar. Approximately 20 cc of frank purulence was drained. A swab of the oropharynx revealed Gram-positive and Gram-negative rods consistent with normal oral flora. No anaerobes were isolated. After drainage, the patient showed significant clinical improvement immediately without need for drain placement. He was discharged on a two-week course of amoxicillin with a steroid taper, warm compress, sialogogues, and pain control (Figures 1–3).

## 3. Discussion

Our patient's presentation was unique in that his abscess extended deeply along the stylomandibular tunnel into the prestyloid parapharyngeal space, whereas most parotid abscesses extend medially to laterally, thus requiring percutaneous surgical management. Additionally, he lacked typical risk factors associated with parotitis and deep parotid abscess formation (he was not diabetic or immunosuppressed and did not have Sjogren's disease, xerostomia, or previous oral surgery).

Accurate statistics on the incidence of deep parotid abscesses are difficult to obtain due to a paucity of literature on the topic—most involving limited case series with less than thirty patients. One retrospective study found parotid abscesses in 1/5 patients with acute parotid disease [2]. The typical presentation of a parotid abscess is swelling in the parotid region with a sudden increase in swelling before seeking care. An acute infection is usually characterized by warm, erythematous skin covering the parotid gland. Infrequently, blood or pus may be seen in the oral cavity. Ipsilateral facial nerve palsy is occasionally but not frequently seen with parotid gland abscess. A 2012 review found only eight previously reported cases of facial nerve palsy secondary to parotid gland abscess [3]. Including that case report, we have noted seven additional cases published in the literature on facial nerve palsy as a result of parotid gland abscess [4–8]. Differential diagnoses usually include various oral neoplasms, primary salivary gland tumors, and inflammatory processes. Unlike neoplasms, infectious causes typically do not cause symptoms for weeks or longer [9]. With peritonsillar infection, cervical and submandibular lymphadenopathy is often prominent but can also be a feature of neoplasm. Additionally, abscesses may form secondary to a malignancy in areas of necrosis—a possible complication in diagnosis [10].

Malnutrition and medications that decrease salivary flow may be predisposing factors for parotitis [11]. In infants younger than two months, parotitis is usually associated with viral infection or transient bacteremia [12]. Diabetes mellitus and Sjogren's syndrome may be predisposing factors for infection and more severe presentation in adults. Poor oral hygiene and tooth extraction history, as well as oral trauma, xerostomia, or ductal obstruction, may also be connected with a higher risk of parotid abscess formation in addition to having a preexisting parotid Warthin's tumor, sialolithiasis or immunosuppression [2, 13].

The parotid gland can become infected via an ascending infection through Stensen's duct, from bacteremia, or even viremia [14]. Many factors may be involved in assisting in the ascension of bacteria through the Stensen's duct. Decreased secretions, especially in a malnourished patient, are a significant factor in bacterial ascension [15]. Ductal ectasis, primary parenchymal involvement, or infection of lymph nodes may play each a role in abscess formation [13]. The deep or superficial lobes of the parotid gland may each give rise to abscess formation. Bacterial infection may lead to inflammatory changes that can lead to abscess formation. The pus may invade the surrounding tissue both deep into the neck and posterior to the auditory canal and can also invade superficially to the face skin. The most common pathogen in acute head and neck abscesses is *Staphylococcus aureus* [16]. However, because the oral cavity is predominated by anaerobic bacteria, anaerobic bacteria are also commonly involved in parotitis. *Streptococci* and Gram-negative bacilli have been recovered [17, 18]. Organisms that are less commonly found include Arachnia, *Haemophilus influenzae*, *Klebsiella pneumoniae*, *Salmonella* spp., *Pseudomonas aeruginosa*, *Treponema pallidum*, *Bartonella henselae*, and *Eikenella corrodens* [19]. Causes of viral infection include rubulavirus, HIV, enteroviruses, Epstein–Barr virus, cytomegalovirus, and influenza [11]. Needle aspiration is the gold standard for diagnosis and treatment in peritonsillar abscess [20]. The common presentation of a peritonsillar abscess consists of a fever, sore throat, and dysphagia. Differentiating symptoms often include trismus and a “hot potato” voice due to pterygoid spasm. The etiology of peritonsillar infection is usually thought to be associated with acute tonsillitis [21].

The parapharyngeal space is a fat-filled area near the parotid gland's deep lobe which consists of a prestyloid and poststyloid space. Abscess extensive into the parapharyngeal space adds significant morbidity as critical neurovascular structures and even the airway may become compromised. The prestyloid space contains the lingual nerve, inferior alveolar nerve, auriculotemporal nerve, and internal maxillary artery. The poststyloid space contains cranial nerves IX, X, XI, and XII as well as the sympathetic chain, internal carotid artery, and internal jugular vein. Vagal dysfunction and cranial nerve deficits, as well as Horner's syndrome, can sometimes be seen in cases where mass effect impinges on the poststyloid space.

The treatment for deep parotid infections generally consists of antibiotics, hydration, and sialogogues to increase movement of saliva. Intravenous antibiotics are recommended, especially for children [22]. Once an abscess has formed, surgery is required to drain the affected space. The choice of the antimicrobial agent varies depending on culture results, but a broad agent that covers aerobic and anaerobic pathogens is generally preferred. To prevent recurrence, the patient should be counseled on good oral hygiene, maintaining sufficient hydration and returning to seek care if fever or swelling reappears.

## 4. Conclusion

We present an unusual case of parotid abscess presenting as a peritonsillar infection that required transoral drainage. Otolaryngologists and emergency room physicians should be aware of the presentation of this abscess in the clinic and operating room due to its mass effect on important structures and the positive impact of early diagnosis and intervention. While a rare diagnosis, parotid abscesses can have significant morbidity and mortality as an untreated parotid abscess can spread quickly through the deep spaces of the head and neck.
